# Integrating animal physiology into the adaptive management of restored landscapes

**DOI:** 10.1007/s00267-023-01800-5

**Published:** 2023-02-13

**Authors:** Emily P. Tudor, Wolfgang Lewandrowski, Sean Tomlinson

**Affiliations:** 1 School of Molecular and Life Sciences, Curtin University, Bentley, WA 6102 Australia; 2 Kings Park Science, Department of Biodiversity, Conservation and Attractions, Kattidj Close, Kings Park, WA 6005 Australia; 3 School of Biological Sciences, University of Western Australia, Crawley, WA 6009 Australia; 4 School of Biological Sciences, University of Adelaide, Adelaide, SA 5005 Australia

**Keywords:** Environmental management, Habitat suitability, Monitoring, Thermal biology, Thresholds, Wildlife conservation

## Abstract

Global-scale ecological changes and intensifying habitat destruction and have caused alarming declines in wildlife populations, resulting in a great need for concerted efforts towards their conservation. Despite this, animals are frequently overlooked in restoration and management initiatives and therefore populations often do not reassemble following disturbance without re-establishing habitat that meets their abiotic and biotic requirements. However, restoration ecologists broadly lack insight into the physiological mechanisms that can govern the responses of fauna to environmental change and management. Therefore, we conducted a literature search for studies reporting a mechanistic understanding of faunal habitat suitability and selection in restored landscapes to deliver an updated perspective on the integration of animal ecophysiology and restoration ecology. Of the 75,442 studies that we identified discussing ecological restoration in the last 50 years, only 8,627 (11.4%) did so in the context of fauna from which 912 studies (1.2%) examined habitat selection, 35 studies (0.05%) integrated physiology and only 15 studies (0.02%) explored thermal biology, despite temperature being one of the most pervasive drivers of physiological functioning. To combat this, we developed a conceptual framework that can guide restoration ecophysiology and promote innovative, multidisciplinary research through an established adaptive management structure. While physiological tools and approaches are currently underutilised in restoration practice, integrating them into ecological restoration, and environmental management more broadly, will offer exciting new opportunities to describe, explain and predict the responses of fauna to environmental change occurring, and that yet to come.

## Introduction

Intensifying anthropogenic pressures have led to global-scale ecological changes that will lead to cascading impacts on landscapes, biodiversity, and human well-being into the future (Crutzen 2006, IPBES 2019). Changing land-use is a leading cause of biodiversity losses with 75% of land having been significantly altered by anthropogenic pressures such as agricultural, industrial, or urban development (IPBES 2019).There has been an average global decline in abundance of 68% across monitored wildlife populations representing 4,392 species in the last 50 years (WWF 2020). Furthermore, without mitigation or reduction of biodiversity and habitat loss, one million species are potentially facing extinction in the coming decades (IPBES 2019). Fortunately, ecological restoration can ameliorate, or even reverse, biodiversity and habitat loss (Suding et al. 2015). The core objective of ecological restoration is to assist the recovery of damaged, degraded or destroyed habitat to a self-sustaining, functioning and resilient ecosystem (Miller et al. 2017). Degradation can result in substantial biological, physical and chemical change (Heneghan et al. 2008). Therefore, mitigation efforts require collaborations between scientific expertise across several ecological disciplines but coupling the expanding knowledge around ecological restoration with practical implementation can be a complex and long-term enterprise (Miller et al. 2017, Bertuol-Garcia et al. 2018). Adding to this challenge is the limited understanding of species’ abiotic and biotic requirements for development, reproduction, and survival, that ultimately drive their successful recovery in restored landscapes.

Topographical, vegetative and hydrological factors frequently dominate the restoration literature and monitoring schemes (McAlpine et al. 2016), while animals are overlooked, owing largely to the assumption that their recolonisation of restored environments occurs without facilitation (Palmer et al. 1997, Hilderbrand et al. 2005, Majer 2009). Animals play critical roles in the delivery of ecological services such as nutrient cycling, pollination, and seed dispersal (Kremen et al. 2007, Noriega et al. 2018). Robust, functional and resilient faunal communities lay the foundations for the continuity of ecosystem function and, consequently, the long-term outcomes for restored ecosystems (Montoya et al. 2012). However, recent meta-analyses conclude that while restoration can improve biodiversity values, fauna compositions and ecological services in degraded landscapes, the resulting communities fail to resemble intact or ‘reference’ ecosystems (Benayas et al. 2009, Crouzeilles et al. 2016, Shimamoto et al. 2018). Additionally, several independent reviews have consistently demonstrated that animals are poorly considered in the restoration ecology literature (Majer 2009, Cristescu et al. 2012, Cross et al. 2019).

To obtain a comprehensive, scalable understanding of fauna recolonisation in restoration, it is necessary to define and explore the potential mechanisms that may explain their responses to environmental change and selection for certain habitats. In this context, mechanism can refer to lower-level biological processes (e.g., respiration) that give rise to higher-level biological processes (e.g., development) as a function of a known environmental variable (e.g., temperature). Understanding causal mechanisms should form the foundation for decision-making and monitoring protocols (Kearney et al. 2009), but the mechanisms underpinning patterns in ecological restoration are often unexplored (Suding 2011, Brudvig 2017). We suggest that understanding the responses of animals to restoration may be best achieved by combining traditional biodiversity surveys with ecophysiology to better describe habitat suitability and selection. This knowledge can be generated by experimental trials under both in situ and ex situ contexts that determine species performance and tolerance to a range of controlled conditions related to biological, chemical, or physical factors in restored landscapes (Cooke et al. 2021). This mechanistic approach can describe the processes underpinning the patterns that emerge throughout restoration, allowing prediction of restoration trajectories to better inform management.

**Fig. 1:**
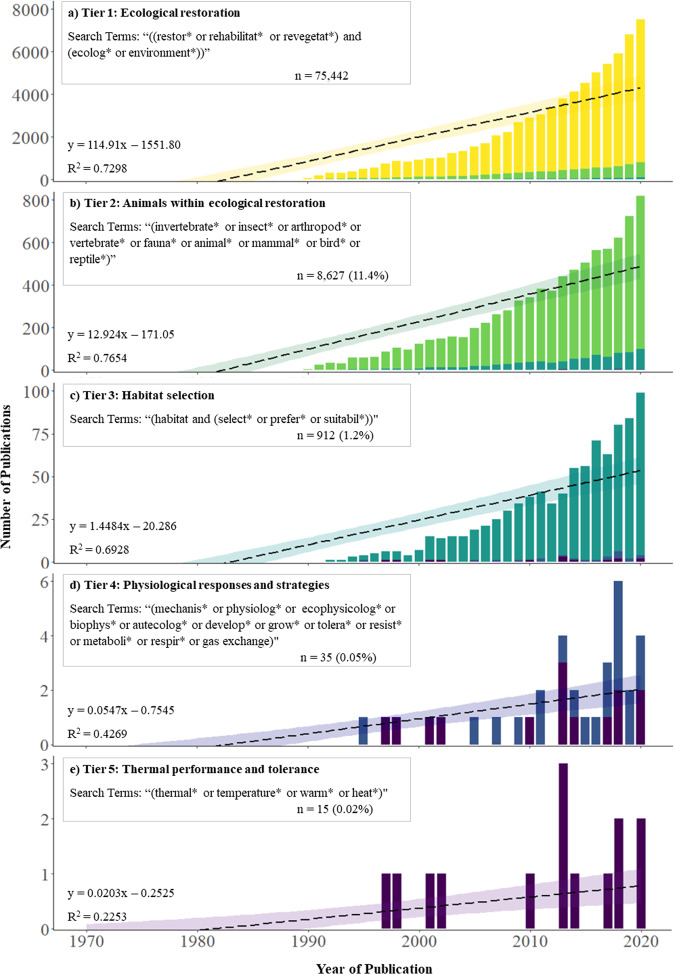
Five-tiered literature search demonstrating (**a**) the development of the restoration ecology literature over the last 50 years (yellow). **b** The representativeness of fauna in the restoration ecology literature (green). **c** The consideration of fauna habitat preference, selection and suitability in restoration ecology (teal). **d** The application of ecophysiological approaches and mechanisms in explaining habitat selection (blue). **e** The assessment of temperature as a driving mechanism for faunal responses to restoration (purple), as an exemplar of a physiologically motivating environmental characteristic. Dashed lines represent linear regression models with colour corresponding banding representing confidence intervals

Our aim was to deliver an updated perspective on the integration of animal ecophysiology into restoration ecology, and specifically to identify ways to inform practice and management. We conducted a literature review of studies that examined ecophysiological mechanisms underpinning habitat suitability and selection for fauna in restored landscapes catalogued by the ISI Web of Science (Core Collection; Last searched 18^th^ May 2021). We refined our search terms across five ‘tiers’ that systematically narrowed our searches from restoration ecology, to studies specifically exploring thermal biology as a key driver of patterns and processes in ecological restoration (Fig. 1). While the physiology of abiotic stress is multifaceted, we narrowed our physiological context to thermal biology as temperature is a fundamental driver of most physiological and biochemical processes in need of greater consideration (Huey 1991, Tuff et al. 2016). We used simple linear regression models to quantify research output over time to compare publication trajectories for each search ‘tier’ and limited our search to studies dealing with terrestrial fauna (i.e., insects, birds, mammals, and reptiles). However, we acknowledge that many aquatic ecosystems also require restoration and believe many concepts discussed here will also translate into current and future freshwater or marine management.

## The place of fauna in ecological restoration

Restoration ecology aims to inform, guide, and support the practice of reinstating functional ecosystems to degraded landscapes (Miller et al. 2017, Wainwright et al. 2018, Tomlinson et al. 2022). Over the last 50 years, restoration ecology has amassed over 75,000 indexed scientific publications (Fig. 1a; Tier 1). Annual research output has grown significantly (Fig. 1a; *t*_1, 49_ = 11.5, *P* < 0.001) with the last decade alone accounting for 54,768 (72.5%) publications (*t*_1,9_ = 18.0, *P* < 0.001), representing a 165% increase in publications compared with the four preceding decades combined (20,674 publications). This substantial increase in research highlights the developing empiricism of restoration science and contribution of restoration research to basic ecological theory (Bradshaw 1983, Jordan et al. 1988, Perring et al. 2015). However, persistent biases in research agendas and monitoring protocols in ecological restoration has been identified repeatedly (Palmer et al. 1997, Hilderbrand et al. 2005). Ecological restoration dominated by consideration of vegetation communities and structure, with comparatively little attention directed towards other elements of ecosystem health and functionality. Arguably one of the most frequently acknowledged shortcomings is failure to consider fauna (Cross et al. 2019).

Fauna are often assumed to return to restored landscapes unaided following the reestablishment of vegetation (Palmer et al. 1997, Hilderbrand et al. 2005). This bias towards the restoration of the floral community overlooks key components of a functioning ecosystem, as restoring vegetation does not always support the return of fauna, nor the associated services they provide (Jones and Davidson 2016). This trend, recognised over three decades ago (Butcher et al. 1989, Majer 1989), is persistent with only 12.4% of papers reporting ecological restoration in the first decade of the 21^st^ century have focused on fauna (Majer 2009), and of these studies, birds typically received the most attention. To examine the place of fauna within terrestrial restoration ecology we refined our first-tier search by the terms “invertebrate* or insect* or arthropod* or vertebrate* or fauna* or animal* or mammal* or bird* or reptile*’, where the asterisk represents a Boolean operator to expand the search to the broadest extent of relevant papers. Publications dedicated to fauna have increased steadily in the last 50 years (Fig. 1b; *t*_1, 49_ = 12.6, *P* < 0.001) and, and the last decade, represents a 139% increase in publications compared to the combined output of the previous four decades (*t*_1,9_ = 12.5, *P* < 0.001). Proportionally, however, the representation by fauna-oriented studies has decreased since the assessment by Majer (2009), and only accounted for 11.4% of the restoration literature that we identified. Despite several reports explicitly advocating for greater consideration of fauna (Majer 2009, Cross et al. 2019), the disparity between animal and plant-oriented studies has increased. This is a substantial oversight considering the critical roles that fauna often play in plant recruitment (Catterall 2018), and ultimately, successful restoration through pollination and seed dispersal. When fauna are considered in restoration, however, assessments tend to be largely descriptive, by associating patterns of taxonomic composition and diversity indices with different ecological states or management practices (Miller et al. 2017). While these approaches provide insights into the spatiotemporal variation of species and community assemblages, correlative associations are limited in their capacity to explain habitat selection and suitability (Weiner 1995, Lawton 1999). Habitat selection is a driving process structuring animal populations in restored landscapes and therefore, successful restoration is dependent upon facilitating suitable habitat that fauna can access and use appropriately (Miller et al. 2017).

Habitat selection by animals is defined by competing environmental costs and benefits to an organism’s performance and fitness (Mayor et al. 2009), and has a profound influence on population dynamics (Pulliam and Danielson 1991), biotic interactions (Martin 2001), and community reassembly (Binckley, Resetarits (2005)). Therefore, it represents a significant, spatially dependent process driving fauna recovery (Hale and Swearer 2017). The number of studies exploring habitat selection have increased steadily since 1970 (Fig. 1c; *t*_1,9_ = 10.5, *P* < 0.001), and significantly over the last decade (*t*_1,9_ = 9.4, *P* < 0.001), but represented less than 1.3% of the broader restoration ecology literature that we identified (Fig. 1a) and only 10.6% of restoration science dedicated to fauna (Fig. 1b). The negligible amount of research dedicated to understanding habitat selection by animals highlights a considerable omission in a field seeking to ameliorate the risks and consequences of habitat loss. Restoration landscapes could be specifically constructed to meet the requirements of animals if the mechanistic drivers of habitat selection were understood (Hale et al. 2019). Yet in many cases, population-level proxies for demography (e.g., abundance) serve as metrics to evaluate the successful or unsuccessful return of fauna to restored habitats. Unfortunately, when such patterns are assessed without reference to function, causality is difficult to infer and if barriers to recovery occur, they are harder to identify and address effectively. In this instance, ecophysiology can help establish links between pattern and process thereby providing critical insight into the mechanisms underpinning species’ responses to ecological restoration. Insights into habitat suitability can characterise physical site conditions and resources necessary for returning functional and sustainable populations and identify potential environmental stressors that may be limiting to restoration success (e.g., temperature and moisture availability).

## The integration of animal ecophysiology and restoration

Cooke and Suski (2008) called for the development and validation of ecophysiological models incorporating laboratory and field approaches to evaluate the relationships between habitat quality, organism performance, and population dynamics. While some studies have recently advanced these efforts by providing theory, model and test in a single restoration context (e.g., Tomlinson et al. 2014, Tomlinson et al. 2017, Tomlinson et al. 2018), our search for terms “mechanis* or physiolog* or ecophysiolog* or biophys*or autecolog* or develop* or grow* or tolera* or resist* or metaboli* or respir* or *gas exchange*” yielded a proportionally low number of publications in the field of ecological restoration. We identified only 209 research reports (0.27%) and only 35 (0.05%) empirical studies incorporated ecophysiological or biophysical assessments of fauna re-assembly in restoration (Fig. 1d). Though high-profile appeals for the integration of restoration and ecophysiology were expressed over a decade ago (Cooke and Suski 2008), and despite the increasingly accessible methodologies available to do so (Cooke and Suski 2008, Tomlinson et al. 2014, Madliger et al. 2018), there has been negligible increase in research productivity in this space following this call to action (Fig. 1d; *t*_1,9_ = 1.8, *P* < 0.10).

Where ecophysiology has been applied to answer questions about fauna in changing environments, it has generally proven to be readily incorporated, transparent, and insightful. For example, high-resolution distribution models that integrate insect thermal constraints have been used to identify how different pollinating guilds (i.e., bees, wasps, and beetles) respond to fragmentation and thermal variability in a restoration landscape (Tomlinson et al. 2018, Tomlinson 2020). Such models can guide the reconstruction of habitat to ameliorate physiological stress and satisfy the energetic requirements of the species. Similarly, the suitability of translocation sites for the critically endangered Western Swamp Tortoise (*Pseudemydura umbrina* Siebenrock, 1901), predicted through biophysical simulation of the species’ thermodynamic niche (Mitchell et al. 2013), identified recipient sites for assisted colonisation in response to climate change. Most recently, such modelling has been used to identify sites where Tasmanian Devils (*Sarcophilus harrisii* Boitard, 1841) could be reintroduced to reinstate predator prey relationships, supress cat predation, and advance otherwise unassisted ecological restoration (Morris et al. 2022). However, models such as these are effectively hypotheses without the validation obtained from field-based studies of free-ranging animals in situ.

The minimal maintenance energetic requirements of an animal can be readily measured and standardised for both ectotherms and endotherms (Withers 1992). The relationship between this fundamental currency of ecology (Kleiber 1961), and abiotic conditions such as ambient temperature, pH, and salinity forms the basis of many of the biophysical models that seek to explain how animals interact with their environment in space and time (Kearney and Porter 2009, Kearney et al. 2010, Kearney et al. 2013). Though rarely explored, when such models have been tested by measuring the cost of living of an animal in situ (e.g., through field metabolic rates; FMR), energetic requirements and expenditure can be remarkably similar to the modelled expectations (e.g., Tomlinson et al. 2017). While some isotopic and telemetric techniques for measuring FMR may not be universally suitable across taxa and environments (Cooke et al. 2004, Cooke 2008, Tomlinson et al. 2014), where feasible, such techniques can present important insights into tolerance constraints on organisms under environmentally relevant conditions. In cases where measurements of true FMR are not feasible, there alternative approaches that provide insight into energetic constraints. Thompson et al. (2018) combined simple ecophysiological experiments with field studies to understand how temperature drives habitat occupancy of two anoles (*Norops humilis* Peters, 1863 and *N. limifrons* Cope, 1862) across forest regeneration stages. Here, thermal constraints of the two species likely caused their avoidance of early stages of restoration. Such approaches are relatively simple to apply broadly and provide mechanistic insights into habitat selection to increase the value of restoration monitoring.

## Using temperature as a driving mechanism in restoration science

The influence of temperature on most physiological and biochemical processes, such as metabolic, growth, and developmental rates, make it one of the most pervasive abiotic drivers of the biology and ecology of both plant and animal taxa (Angilletta 2006, Kooijman and Kooijman 2010, Gilbert et al. 2014, Buckley et al. 2015). Consequently, calls to explore the thermal drivers of habitat selection are not new (Huey 1991), and have been reiterated recently (Tuff et al. 2016, Tomlinson et al. 2018, Garcia and Clusella-Trullas 2019). Therefore, we further refined our search to “thermal* or temperature* or warm* or heat*”, identifying only 15 studies (0.02% of the restoration ecology literature) that incorporate thermal physiology into fauna reassembly research (Fig. 1e). There has been no significant increase in publications in the last decade, contrasting with the trends observed in the broader literature (Fig. 1a–c). To our knowledge, thermal performance has not been used to explain patterns of population or community reassembly in the context of ecological restoration. By examining thermal performance, restoration ecologists may be able to better predict potential demographic bottlenecks and forecast which species will persist, decline, or drop out of the modified systems, and those that have the capacity to recover with ongoing restoration management.

At large scales, climate change has prioritised research into temperature-driven contributions to conservation priorities (Tylianakis et al. 2008, Gilman et al. 2010, Lister and Garcia 2018). However, habitat degradation and subsequent restoration can cause localised shifts in the thermal environment (Meyer et al. 2001). Depending on the context and stages of ecological restoration some sites can be warmer, more exposed, and more desiccating environments (Cross et al. 2020), or they can be cooler and less suitable for native animals to manage their thermal biology (Garcia and Clusella-Trullas 2019). Consequently, the recovery of fauna in a restoration landscape depends, in part, on the ability of individuals to tolerate novel temperature regimes arising from degraded and restored ecosystems (Meyer and Sisk 2001, Meyer et al. 2001). Therefore, it is essential for scientists and practitioners to be aware of the potential thermal implications associated with habitat degradation, restoration, and management to effectively ameliorate thermal stress and maximise thermal suitability for wildlife populations. Integrating ecophysiology with thermal biology and other disciplines, such as community and population ecology, offers exciting new pathways to increase restoration success by developing mechanistic insights to guide the design, implementation and on-going management of ecological restoration.

## Experimental adaptive management: The opportunity to integrate ecophysiology into restoration ecology

While the importance of interdisciplinary research integrating physiology and restoration ecology has been long insisted upon (Cooke and Suski 2008), translating ecophysiological techniques into tools for practical ecological restoration remains a major challenge. However, criticism has been directed at ecophysiologists for primarily communicating their findings to other scientists through peer-reviewed literature (Cooke and O’Connor 2010). This risks the field of physiology becoming an echo-chamber, and the value of the research being poorly communicated to practitioners (Cooke and O’Connor 2010, Seavy and Howell 2010). Conversely, the practical outcomes of restoration projects are often not published in peer-reviewed journals, restricting feedback from practitioners to the scientific community (Sunderland et al. 2009). This also restricts the communication of lessons learned only to local groups, regardless of the global challenge that ecological restoration represents. Practitioners have also been criticised for not seeking and using the most appropriate, evidenced-based support for their management actions (Sutherland et al. 2004, Cooke and O’Connor 2010). As a science, however, ecophysiology has been criticised for operating at inadequate biological, spatial and temporal scales (Cooke and O’Connor 2010), and of having objectives that do not align with the knowledge needs of practitioners and decision-makers (Cooke and Suski 2008, Cooke and O’Connor 2010). We suggest that increased communication between both physiologists and ecologists, and between science and practice can be beneficial to all parties. In this regard, practitioners and ecologists should collaborate further and facilitate bi-directional flows of knowledge to maximise restoration success (Baker et al. 2014, Young et al. 2014, David et al. 2016).

**Fig. 2:**
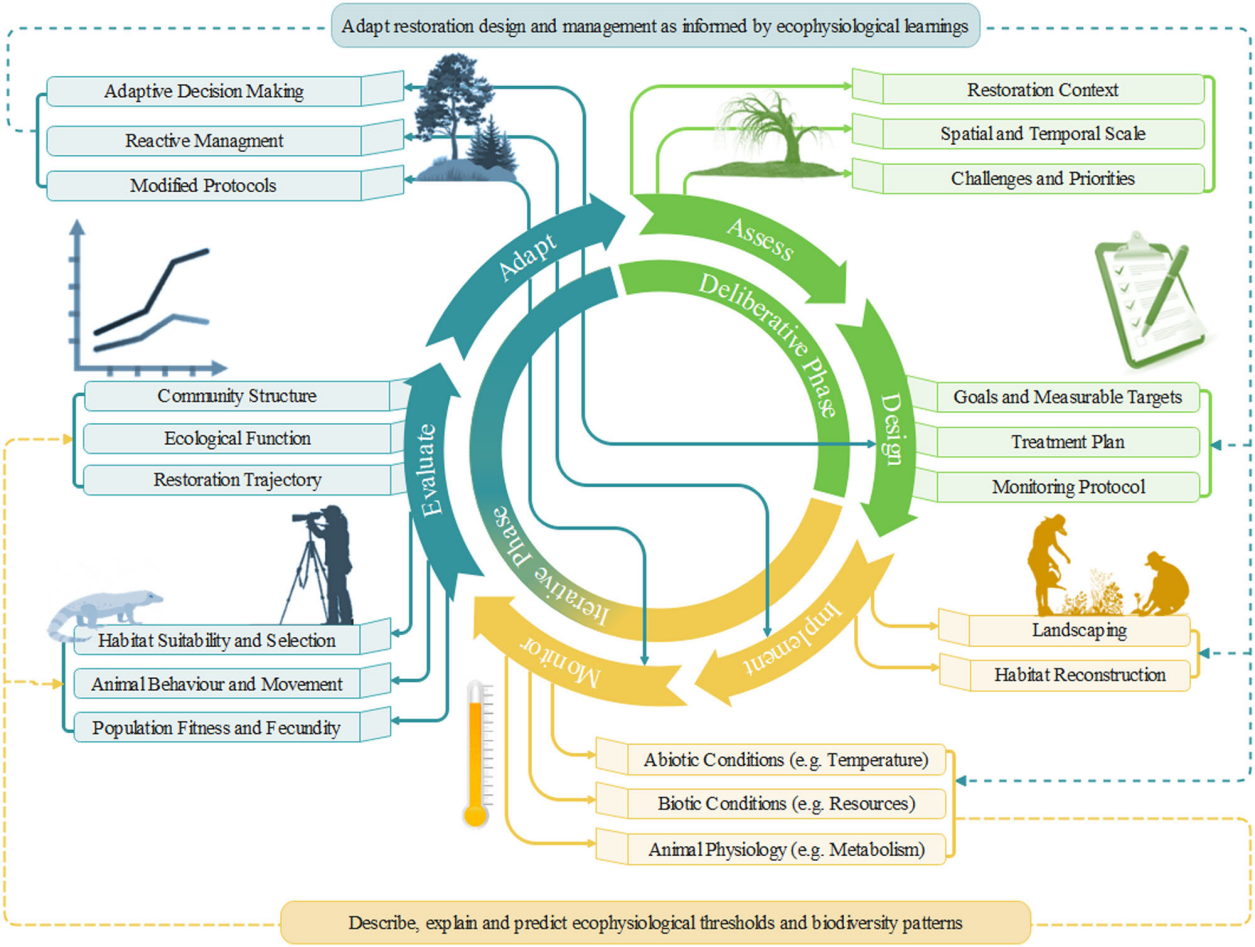
Schematic overview representing how animal physiology can be used across an adaptive management cycle to describe, predict, and explain organism responses to ecological restoration, contribute to the evaluation of restoration trajectories and inform management actions to feed back into the design, implementation, and ongoing management of ecological restoration in a flexible, iterative process of decision making and knowledge acquisition

One way to foster greater collaboration between animal ecophysiology and restoration ecology is to embed ecophysiology into adaptive management (Fig. 2). Adaptive management is a structured, cyclical process of decision-making that accounts for change and uncertainty in a “learning by doing” fashion where actions are undertaken iteratively to minimise uncertainty and improve upon previous efforts (Williams 2011, McDonald et al. 2016). Adaptive management is regarded as the standard approach to ecological restoration (McDonald et al. 2016, SERA 2017), and should be designed with the input of both researchers and practitioners (Taylor et al. 1997, Morghan et al. 2006). Adaptive management generally conducts regular monitoring and evaluation to assess whether current management actions are achieving set goals and modifying these actions to address any shortcomings identified and minimising uncertainty through ongoing acquisition of knowledge (Fig. 2; Murray and Marmorek 2003). However, the implementation of an adaptive management framework can be optimised with experimental designs that test explicit hypotheses (Bormann et al. 2007, Williams 2011).

Species-specific ecophysiology and site-specific conditions (and manipulation of such) have high applicability in the design, implementation, monitoring and adaptive management of ecological restoration (Fig. 2; Tomlinson et al. 2022). While traditional biodiversity monitoring (e.g., species richness, abundance and composition) can describe patterns of spatial and temporal variation, they are limited in their capacity to provide causal interpretation of restoration successes and failures (Lawton 1999, Verberk et al. 2013). Integrating experimental physiology into restoration management can delineate cause-and-effect relationships between organism performance (e.g., developmental rate, metabolic rate, reproductive output) and limiting environmental factors and predict the responses of fauna to environmental change or management practices (Cooke and Suski 2008, Tarszisz et al. 2014). For example, using ecophysiological approaches to assess pre-disturbance conditions of an ecosystem (e.g., before mining or clearing) can establish a reference system that maximises habitat suitability for returning fauna or the suitability of translocation sites as a mitigation strategy (“Assess”; Fig. 2; Tarszisz et al. 2014). This information can then be used to design restoration goals and treatment plans that maximise the necessary resources and physical characteristics to support the physiological and energetic constraints of key functional groups and target taxa (“Design”; Fig. 2). For example, initial seed blends may be selected to provide greater nutritional and provisioning resources for keystone pollinators (Tomlinson et al. 2018), or landforms may be specifically designed to maximise microhabitat variability, creating different niches across a restoration landscape (“Implement”; Fig. 2; Milling et al. 2018). Ecophysiological insight can also inform management actions that increase barriers to fitness to minimise species invasion or optimise biological control practices (Schmitz and Barton 2014, Tougeron et al. 2016).

Trigger points and clear measurable indicators can also be established empirically through physiological metrics such as tolerance thresholds or performance parameters. Regular site monitoring informed by these metrics can establish whether restoration actions achieve restoration goals by comparing changes in animal physiology from an established baseline or comparing mean responses between restored and ‘reference’ populations (“Monitor”; Fig. 2; Madliger et al. 2018). The inclusion of stress, nutritional and reproductive physiology into monitoring protocols provides valuable insights into the synergistic links between animal behaviour, movement and ultimately habitat selection restoration initiatives (Tarszisz et al. 2014, Tomlinson et al. 2018, Tomlinson 2020). However, biotelemetric approaches (Cooke et al. 2004), in conjunction with measures of traditional biodiversity values (e.g., species abundance, richness, and diversity) or functional responses (e.g., fecundity or behaviour), can greatly enhance our understanding of the patterns of reassembly that emerge in restored landscapes (Seebacher and Franklin 2012). Doing so can provide mechanistic perspectives to traditional monitoring outcomes and determine whether restoration practice needs to be adjusted.

Coupling in situ biodiversity surveys, ex situ physiological measurements and landscape ecology can deliver unparalleled insight into which restoration activities can maximise habitat suitability, organism performance and ultimately, function (“Evaluate”; Fig. 2). Ecophysiology can assist in the evaluation of restoration practices that support the transition of degraded sites towards a reference target, and those that do not, while delivering a unique capacity to predict future responses by drawing cause-and-effect relationships (Cooke and Suski 2008). For example, comparative behavioural, reproductive, and developmental physiology can be conducted ex situ and in situ to explain variation in physiological performance relative to the biotic and abiotic conditions of different successional ages, structure, or quality (Tudor 2021). Such insights can *describe* ecophysiological variation, *explain* emerging patterns of biodiversity and *predict* future responses to changing environments. Collectively, ecophysiology can provide evidence-based guidance to evaluate the consequences and uncertainties of management actions and feed into the adaptive decision making of how management can be modified to improve future outcomes. Following this, ecophysiology can iteratively feed back into the design, implementation and monitoring of restoration in such a way that balances between the acquisition of knowledge to minimise uncertainties to improve future outcomes and achieving the best short term results based on current knowledge (“Adapt”; Fig. 2; Murray and Marmorek 2003).

While such high-resolution physiological data are not essential for all species, applying this framework to key focal groups, such as keystone species or ecosystem service providers, provides a strategic avenue for maximising physiological insight that can be used to guide restoration initiatives (Tomlinson et al. 2022). The adaptive management schema that we have developed here is not necessarily a new framework; it instead identifies how to incorporate novel ecophysiological measurements into an established management structure to gain new insights into ecological restoration. While this framework will not be enough to close the gap between science and practice, it offers a tangible tool to foster the integration between the two emerging fields of restoration ecology and conservation physiology. This integrative, interdisciplinary approach may have been an overlooked element in allowing ecophysiology to play a substantial and informative role in ecological restoration, and we are hopeful that applying this framework to other ecophysiological traits will offer exciting new pathways to increase restoration success and respond to the challenges that we have set for ourselves in the Anthropocene.

## Conclusions

Our literature review showed that, while restoration ecology is a rapidly growing field of research (Fig. 1a), fauna remain poorly integrated (Fig. 1b). In fact, fauna are considered proportionally less than they were a decade ago (Majer 2009). Despite the intrinsic value in empirically describing the interactions between fauna and restoration practices, physiological studies are rare in restoration ecology, and responses to the most pervasive abiotic driver, temperature, are hardly considered at all (Fig. 1e). This is a substantial oversight given that preference or avoidance of different habitats is often motivated largely by physiological constraints and that physiological requirements can profoundly alter the interaction between animals and their biotic niche (Nowakowski et al. 2018). Ecophysiological approaches exist to bridge this knowledge gap and they can be rapidly integrated into any restoration context to help understand complex organism-environment dynamics. Collaborations between restoration practitioners and scientists should aim to not only optimise protocols to better describe, explain and predict responses of biological systems, but to also engage with the iterative improvement of long-term management and research to maximise the value of restored landscapes for faunal communities.
